# Posttraumatic Stress Symptoms in Patients on Ventricular Assist Device Support: Something to Worry About?

**DOI:** 10.1111/ctr.70556

**Published:** 2026-05-18

**Authors:** Christiane Kugler, Sadhbh Byrne, Hannah Spielmann, Fabian Richter, Sandra Semmig‐Könze, Christine Spitz‐Koeberich, Wolfgang Albert, Katharina Tigges‐Limmer, the Selma Study Group

**Affiliations:** ^1^ Faculty of Medicine University of Freiburg, Institute of Nursing Science Freiburg Germany; ^2^ Department of Cardiothoracic and Vascular Surgery German Heart Center Charité Berlin Germany; ^3^ Charité – University Medicine Berlin, Corporate Member Free University of Berlin and Humboldt‐University Berlin Berlin Germany; ^4^ Leipzig Heart Center Leipzig Germany; ^5^ University Heart Center Freiburg ‐ Bad Krozingen, Faculty of Medicine, Medical Center ‐ University of Freiburg Freiburg Germany; ^6^ Heart and Diabetes Center North‐Rhine Westphalia University Hospital of the Ruhr, University Bochum Bad Oeynhausen Germany

**Keywords:** heart‐related trauma, posttraumatic stress symptoms, ventricular assist device

## Abstract

**Background:**

Psychosocial outcome in ventricular assist device (VAD) patients continues to merit evaluation including posttraumatic stress symptoms (PTSS).

**Methods:**

A cross‐sectional multicenter study collected data from 265 VAD patients, mean age was 59 ± 11yrs, 88% (*N* = 234) were male. Standardized PRO measures were applied; PTSS was assessed with the Breslau scale, supplemented by open‐ended questions on “any traumatizing life events”.

**Results:**

The weighted mean prevalence for patients reporting “any traumatizing event” was 43.0% (95% CI 37.0–49.2); prevalence for PTSS was 3.8% (95% CI 1.82–6.83). Traumatizing events were categorized into non‐heart‐related versus heart‐related (both *n* = 57; 50%); heart surgery (*n* = 16) was most prominent. Oral intake of prescribed psychotropics (12.5%) was significantly higher in those with non‐heart‐related events (28.1% vs. 7.0%; *p* = 0.006). Female gender was associated with an OR of 1.703 (95%CI: 1.217–2.382; *p* = 0.002) for HADS_depression_ and oral psychotropics (OR 6.623, 95%CI: 2.009–21.830; *p* = 0.002). HRQoL was lower for those on psychotropics (*p* = 0.001). Patients who had experienced a trauma showed no differences in PTSS (*p* = 0.771), anxiety (*p* = 0.774), depression (*p* = 0.565), and HRQoL (*p* = 0.113).

**Conclusion:**

A substantial proportion of VAD patients may have experienced traumatizing events; heart surgery was reported most prominently, may diminish the success of implantation, lead to PTSS, and warrants routine clinical screening of patients following VAD implantation.

**Abbreviations**
CI: confidence interval
HADS: hospital anxiety and depression scale
HRQoL: health‐related quality of life
ICU: intensive care unit
KCCQ: Kansas city cardiomyopathy questionnaire
OR: odds ratio
PHQ‐9: patient health questionnaire, 9‐item version
PRO: patient‐reported outcome
PTSS: posttraumatic stress symptoms
VAD: ventricular assist device

## 1 Background

Recent data show an alarming projected increase of heart failure patients of 34% in upcoming decades in the western world [1, 2]. For patients, where conservative treatment options have been exhausted, ventricular assist devices (VADs) become an established treatment option to change the natural trajectory of heart failure [1, 3]. However, this treatment modality may inflict psychosocial comorbid conditions, namely symptoms of anxiety, depression, and posttraumatic stress (PTSS), which have been shown to predict postimplantation outcomes [4, 5, 6, 7]. Although some data suggest that anxiety and depression symptoms may decrease over time [8], VAD patients bridged to transplant and those on destination therapy share the burden of uncertainty and the dependency on a pump on a day‐to‐day basis [9, 10].

Patients may face an increased risk for the development of PTSS as a result of an episode of the acute, severe or even life‐threatening disease, its diagnosis and treatment procedures, and/or a significant amount of time spent in the intensive care unit (ICU) [11, 12, 13]. A recent meta‐analysis by Parker and associates [14] assessed the prevalence of PTSS and pooled data from over 3,400 general critical illness survivors. They calculated a point prevalence of substantial PTSS ranging between 25% and 44% at 1–6 months following a prolonged ICU stay [14]. Moreover, a Dutch study led by Boer and colleagues [11] assessed factors associated with increased PTSS in a multivariate regression model and identified younger age, length of ICU stay, and having traumatic memories of the ICU or hospital stay as contributing to the risk of PTSS.

End‐stage heart failure, its associated invasive diagnostic tests and treatment, VAD implant surgery, ICU stay, ventilation, and pain may all have potential to lead to psychosocial comorbid conditions, namely to PTSS. However, patients may have experienced PTSS due to another lifetime event completely unrelated to their heart disease. Estimates of lifetime PTSS prevalence in community samples range between 5% and 6% for men and 10% and 12% for women [13]. Therefore, understanding the epidemiology of PTSS is essential in order to prevent or treat this risk factor for adverse outcomes early on [14]. Thus, our study was undertaken to (1) determine the occurrence of psychosocial comorbid conditions defined as symptoms of anxiety, depression and PTSS in VAD patients, (2) differentiate between heart‐related versus non‐heart‐related PTSS events according to the patients’ self‐report, (3) examine the interrelationship between those psychosocial comorbid conditions, and (4) identify independent determinants of psychosocial comorbid conditions post‐VAD implantation.

## 2 Materials and Methods

### 2.1 Design and Setting

A cross‐sectional design served for the study purposes. Patients on ongoing continuous‐flow VAD support were recruited as part of a national multicenter study at established heart centers (NCT04234230); a detailed description is provided in the study protocol [15]. The protocol was approved by the institutional review board of the host institution (IRB‐No. 304/19); participating centers’ review boards confirmed this approval before data collection initiation. Study procedures were in concordance with the Helsinki Declaration and the European data protection regulation [16]. Results were reported in accordance with the STROBE guidelines [17].

### 2.2 Study Participants

**FIGURE 1:**
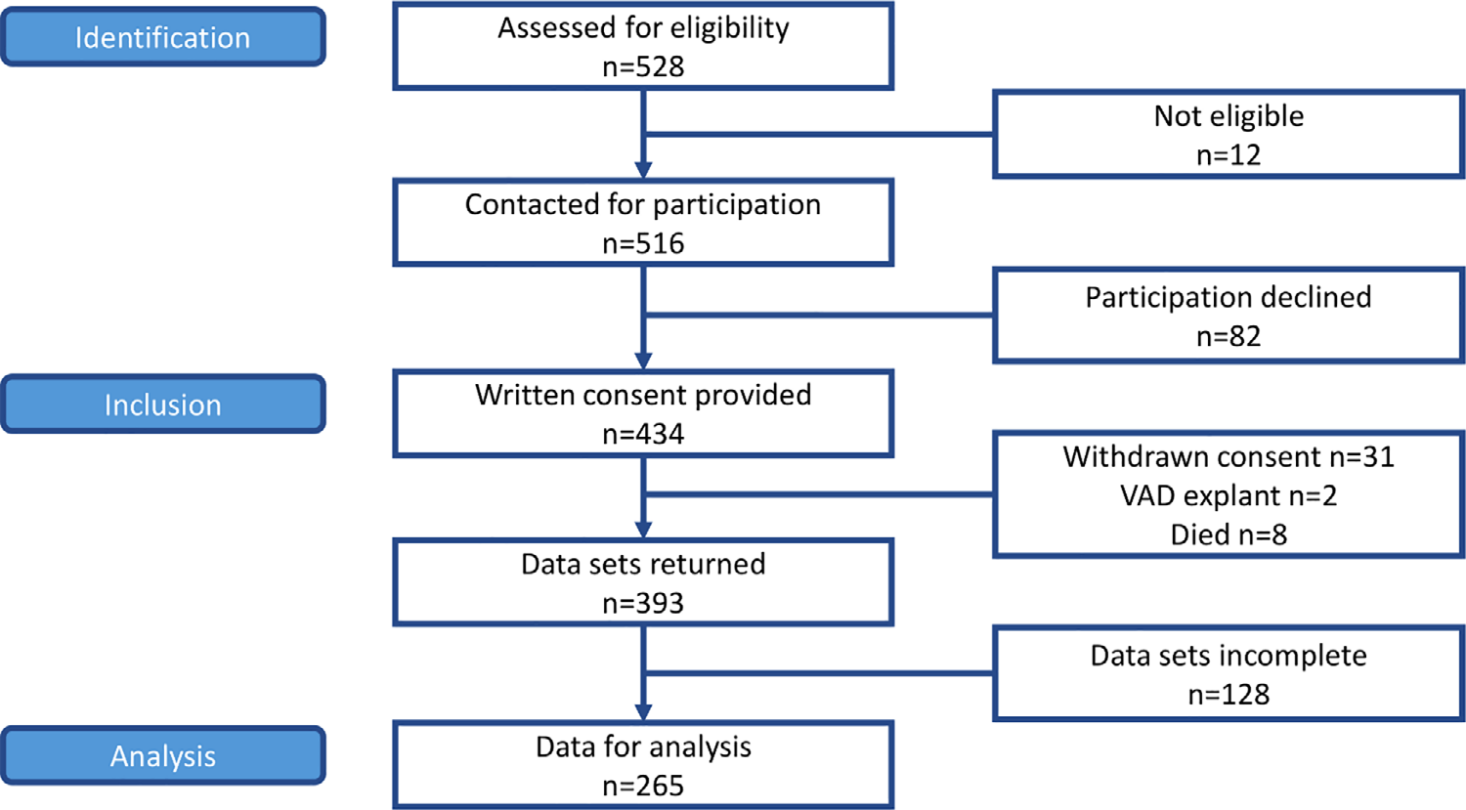
Study inclusion flowchart.

Selection of participants was performed in patients who fulfilled the following inclusion criteria: clinically stable outpatient on ongoing VAD support (3–36 months post‐implant), age 18 or older, cognitive and language capability to participate in a patient‐reported outcome (PRO) study [15]. Figure 1 depicts the study flowchart. The initial sample consisted of 393 patients; of those 265 returned completed data on posttraumatic stress symptoms (PTSS), leading to a 67.4% response rate.

### 2.3 Instruments and Measures

#### 2.3.1 PRO Measures

A questionnaire package with standardized validated instruments was utilized for this study. Data collection was performed during regular outpatient clinical visits and included the opportunity to return the packages in a pre‐stamped and pre‐addressed envelope to the study center. The following PRO instruments in psychometrically tested German versions were included:

##### 2.3.1.1 Breslau 7‐item PTSS scale

The Breslau seven‐item PTSS scale represents a generic screening measure for symptoms of PTSS developed by Breslau and associates [18], a validated German version was published by Siegrist and Maercker [19]. The scale is unidimensional, has a one‐month recall period, and comprises seven items that cover symptoms of PTSS, such as re‐experiencing, avoidance, and hyperarousal. The sum score ranges between 0 and 7, higher scores indicate higher symptom burden. In cases, where a cut‐off score of ≥4 positively rated items are assessed, screening for a diagnosis of PTSD is recommended [18, 19]. The seven‐item scale is preceded by an open‐ended question asking whether this person experienced and remembers any subjectively perceived serious or traumatizing life event, and if so, what this was. The open‐ended question provides no time frame for recall, allowing respondents to reflect on any relevant period. The open‐ended question was analyzed to generate categories of responses based on content analysis methodology. Non‐responses and responses ‘no event occurred’ were categorized as ‘no event reported’ by the authors. Two nominal follow‐up questions (yes/ no) ask for feelings of helplessness and severe anxiety/ horror related to that event. Patients were asked to report the presence of each symptom (yes/ no) for the last four weeks. The developers reported an 80% sensitivity and 97% specificity for this PRO measure [18].

##### 2.3.1.2 Hospital anxiety and depression scale (HADS)

The HADS [20] is an established generic screening tool to assess symptoms of anxiety and depression along two scales with seven items each. Scale scores range from 0 to 21, higher scores indicate more severe symptoms of anxiety and depression [20].

##### 2.3.1.3 Patient health questionnaire‐9 (PHQ‐9)

The PHQ‐9 [21] represents a screening tool for symptoms of depression using nine items on a four‐point Likert scale; scores range from 0 to 27 with higher values reflecting more symptoms of depression [21].

##### 2.3.1.4 Kansas city cardiomyopathy questionnaire (KCCQ)

The KCCQ [22] represents a widely used disease‐specific instrument to assess health‐related quality of life (HRQoL) in chronic heart failure populations. The instrument comprises 23 items; following transformation, scale scores range from 0 to 100, with zero representing the worst possible HRQoL and 100 the best possible HRQoL. Scale scores are transformed to the following summary scores: functional status, clinical status, and an overall summary score [22].

### 2.4 Other Measures

Additional measures were retrieved from the patient charts with consent from those participating and included the following variables: relevant VAD‐related adverse events (all yes/ no; all within the last 3 months before data collection), including driveline exit site infections, bleeding events, and thromboembolic events, as well as prescription of psychotropic medications. Demographic data were collected as part of the questionnaire package.

### 2.5 Statistical Analysis

Descriptive statistical analyses were performed based on variable level as appropriate. Prevalence for PTSS was presented in percentage and 95% confidence interval (95% CI). Memories about subjectively perceived serious or traumatizing life events were captured in an open‐ended format, categorized using content analysis methods, and grouped into the following three categories: ‘no event reported’, ‘heart‐related event reported’, and ‘non‐heart‐related event reported’ by two persons of this work group (CK, SB). Inconsistencies were discussed and resolved by consensus. Group comparisons were performed to identify differences between the ‘heart‐related’ versus ‘non‐heart‐related’ event group using the non‐parametric data format and calculated with Mann‐Whitney‐U tests. Pearson's chi‐squared (*χ* ^2^) test was used to test for relationships between categorical variables and Fisher's exact test was used where statistical testing requirements were not fulfilled. The Student's *t*‐test was used to test for differences in mean scores across interval scale variables, using the Mann‐Whitney‐U test in cases of non‐normality and ordinal‐scaled variables. Binary logistic regression models with block‐wise selection and backward likelihood method were used to assess the influence of gender and use of oral psychotropics as predictors of psychosocial comorbid conditions. Missing cases were handled using pairwise deletion. Statistical analyses were conducted using IBM SPSS Statistics Version 30. The level of significance for two‐tailed testing was set at 0.05.

## 3 Results

### 3.1 Sample Characteristics

The sample comprised 265 outpatients on ongoing VAD support with a mean (±SD) age of 59 (±11), the majority of 88.3% being men, 57.0% were bridged to transplant, 23.4% were on destination therapy, and 17.4% other implant reasons excluding missing values (2.2%). Table 1 depicts relevant characteristics of the overall sample, those with no reported PTSS related events, and differentiates further between heart‐related versus non‐heart‐related PTSS events according to the patients’ self‐report. Oral intake of prescribed psychotropics (12.5% overall) was significantly higher in those with non‐heart‐related events in comparison to the sub‐sample with heart‐related events (28.1% vs. 7.0%; *p* = 0.006).

**TABLE 1:** Sample characteristics (*n* = 265).

	Total	No event reported	Non‐heart‐related event	Heart‐related event	
	*N* = 265	*N* = 151	*N* = 57	*N* = 57	*p*‐value^*^
**Sex**					
Male	234 (88.3%)	136 (90.1%)	47 (82.5%)	51 (89.5%)	0.419
Female	31 (11.7%)	15 (9.9%)	10 (17.5%)	6 (10.5%)	
**Age**					
Mean (SD)	59.1 (±11.0)	62.0 (±10.1)	59.3 (±12.3)	57.7 (±11.6)	0.483
**Living status**					
Alone	70 (26.4%)	36 (23.8%)	18 (31.6%)	16 (28.1%)	0.838
Not alone	193 (72.8%)	113 (74.8%)	39 (68.4%)	41 (71.9%)	
Missing	2 (0.8%)	2 (1.3%)	—	—	
**Education**					
High school degree	98 (37.0%)	56 (37.1%)	19 (33.3%)	23 (40.4%)	0.553
College degree	109 (41.1%)	68 (45.0%)	20 (35.1%)	21 (36.8%)	
University degree	50 (18.9%)	21 (13.9%)	17 (29.8%)	12 (21.1%)	
Missing	8 (3%)	6 (4.0%)	1 (1.8%)	1 (1.8%)	
**Employment status**					
Employed	28 (10.6%)	16 (10.6%)	6 (10.5%)	6 (10.5%)	1.000
Retired due to illness/ job seeking	144 (54.3%)	79 (52.3%)	33 (57.9%)	32 (56.1%)	
Retired due to age	82 (30.9%)	48 (31.8%)	18 (31.6%)	16 (28.1%)	
Missing	11 (4.2%)	8 (5.3%)	—	3 (5.3%)	
**Time since implantation**					
Year 1: 3–12 months	108 (40.8%)	65 (43.0%)	21 (36.8%)	22 (38.6%)	0.504
Year 2: 13–24 months	90 (34.0%)	49 (32.5%)	19 (33.3%)	22 (38.6%)	
Year 3: 25–36 months	66 (24.9%)	36 (23.8)	17 (29.8%)	13 (22.8%)	
Missing	1 (0.4%)	1 (0.7%)	—	—	
**Type of surgery**					
Elective	204 (77.0%)	121 (80.1%)	45 (78.9%)	38 (66.7%)	0.257
Emergency	48 (18.1%)	23 (15.2%)	10 (17.5%)	15 (26.3%)	
Missing	13 (4.9%)	7(4.6%)	2 (3.5%)	4 (7.0%)	
**Intake of psychotropics**					
Yes	33 (12.5%)	13 (8.6%)	16 (28.1%)	4 (7.0%)	**0.006**
No	231 (87.2%)	137 (90.7%)	41 (71.9%)	53 (93.0%)	
Missing	1 (0.4%)	1 (0.7%)	—	—	
**Type of psychotropics**					
Anti‐depressants	28 (10.6%)	11 (7.3%)	14 (24.6%)	3 (5.3%)	0.509
Neuroleptics	3 (1.1%)	—	2 (3.5%)	1 (1.8%)	
**Adverse events**					
Yes	156 (58.9%)	60 (39.7%)	34 (59.6%)	32 (56.1%)	0.850
No	108 (40.8%)	90 (59.6%)	23 (40.4%)	25 (43.9%)	
Missing	1(0.4%)	1 (0.7%)	—	—	
**Type of adverse event**					
Thrombo‐embolic‐neuro	21 (7.9%)	10 (6.6%)	7 (12.3%)	4 (7.0%)	0.528
Bleeding on the driveline	29 (10.9%)	18 (11.9%)	4 (7.0%)	7 (12.3%)	0.528
Infection on the driveline	83 (31.3%)	50 (33.1%)	16 (21.8%)	17 (29.8%)	1.000
**Implantable cardioverter defibrillator (ICD)**					
Yes	158 (59.6%)	84 (55.6%)	38 (66.7%)	36 (63.2%)	0.845
No	105 (39.6%)	65 (43.0%)	19 (33.3%)	21 (36.8%)	
Missing	2(0.8%)	2 (1.3%)	—	—	

* *P*‐values refer to differences in heart‐related and non‐heart‐related event groups.

### 3.2 Occurrence of Psychosocial Comorbid Conditions

#### 3.2.1 Post‐Traumatic Stress Symptoms

In this sample, 114 patients confirmed that they had experienced and remembered subjectively perceived serious or traumatizing life events. They included the precise names or descriptions for those specific events in an open‐ended text field. This represents a prevalence of 43.0% (95%CI: 37.0%–49.2%). By content analysis these events were categorized into events being non‐heart‐related (*n* = 57; 50%) versus heart‐related (*n* = 57; 50%) (Table 2).

**TABLE 2:** Content‐based categories of traumatizing life events.

Non‐heart‐related event^*^	*N* = 57 (50%)	Heart‐related event^*^	*N* = 57 (50%)
Experienced violence	*n* = 14	Heart surgery/ VAD implantation	*n* = 16
Death/ serious disease of a family member	*n* = 13	Myocardial infarction	*n* = 13
Serious somatic disease (non‐heart self)	*n* = 10	Defibrillation	*n* = 8
Divorce/ separation	*n* = 7	Acute delirium post‐op	*n* = 5
Mental disease (self)	*n* = 6	Prolonged ICU/ hospital stay	*n* = 4
Others	*n* = 20	Heart failure	*n* = 4
		Others	*n = 14*

* Patients were asked to report on “any traumatizing life event” by “naming” the event in an open‐ended text field; more than one name/ description was possible; categories performed based on content‐analysis technique.

**FIGURE 2:**
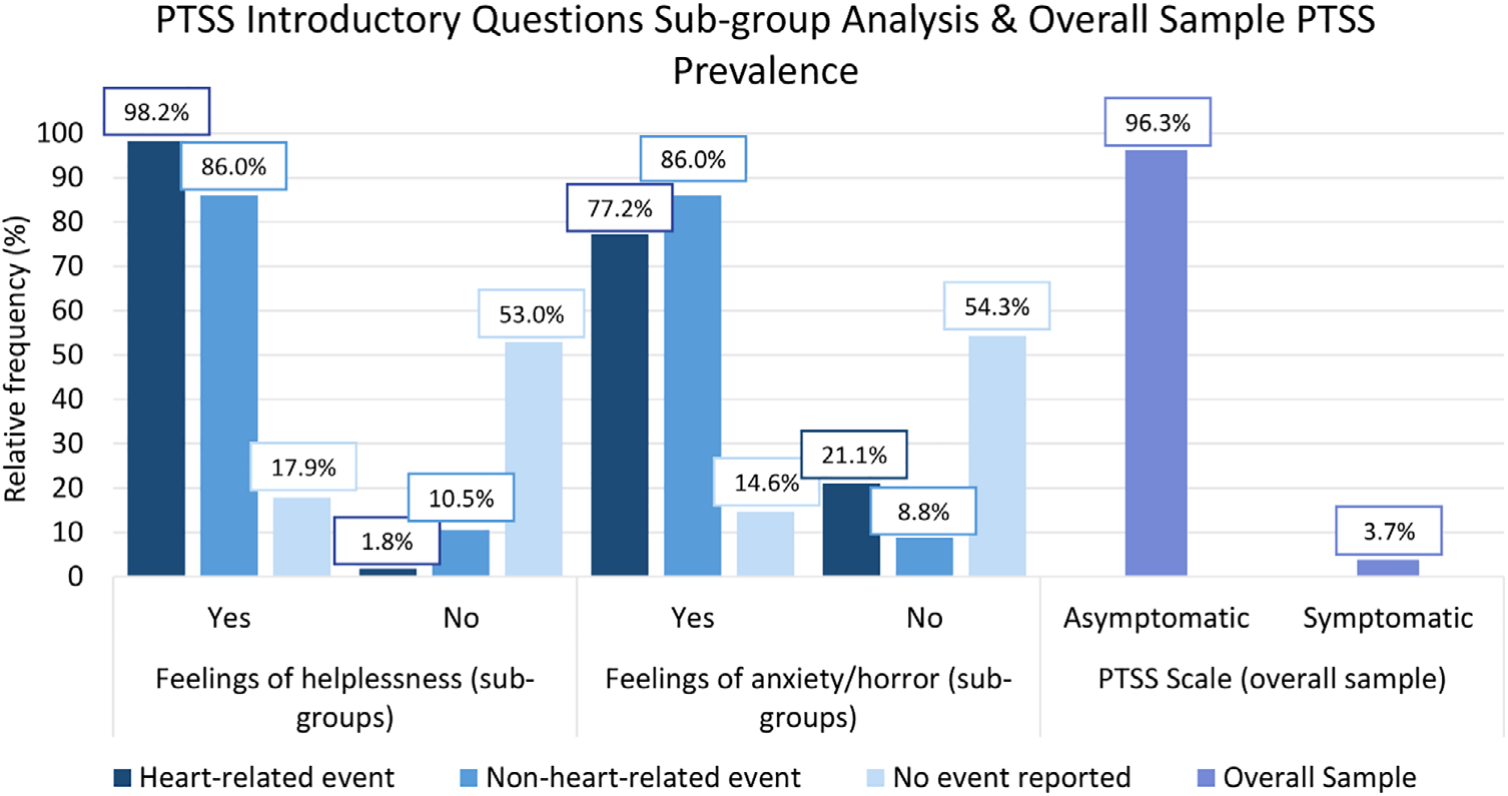
Introductory questions 1–2 Breslau scale: “During the perceived event, did you experience…?”. *Percentages smaller than 100% refer to missing values.

Feelings of helplessness were reported by 49.8% of the overall sample, and severe anxiety/ horror related to that event by 43.4% of the overall sample. In addition, sub‐group analysis of the introductory questions by event type are displayed in Figure 2.

Screening for PTSS symptoms was based on the 7‐item Breslau scale and revealed PTSS occurrence in 10 patients (3.7%, 95%CI: 1.8%–6.8%) (Figure 2).

#### 3.2.2 Anxiety and Depression

**FIGURE 3:**
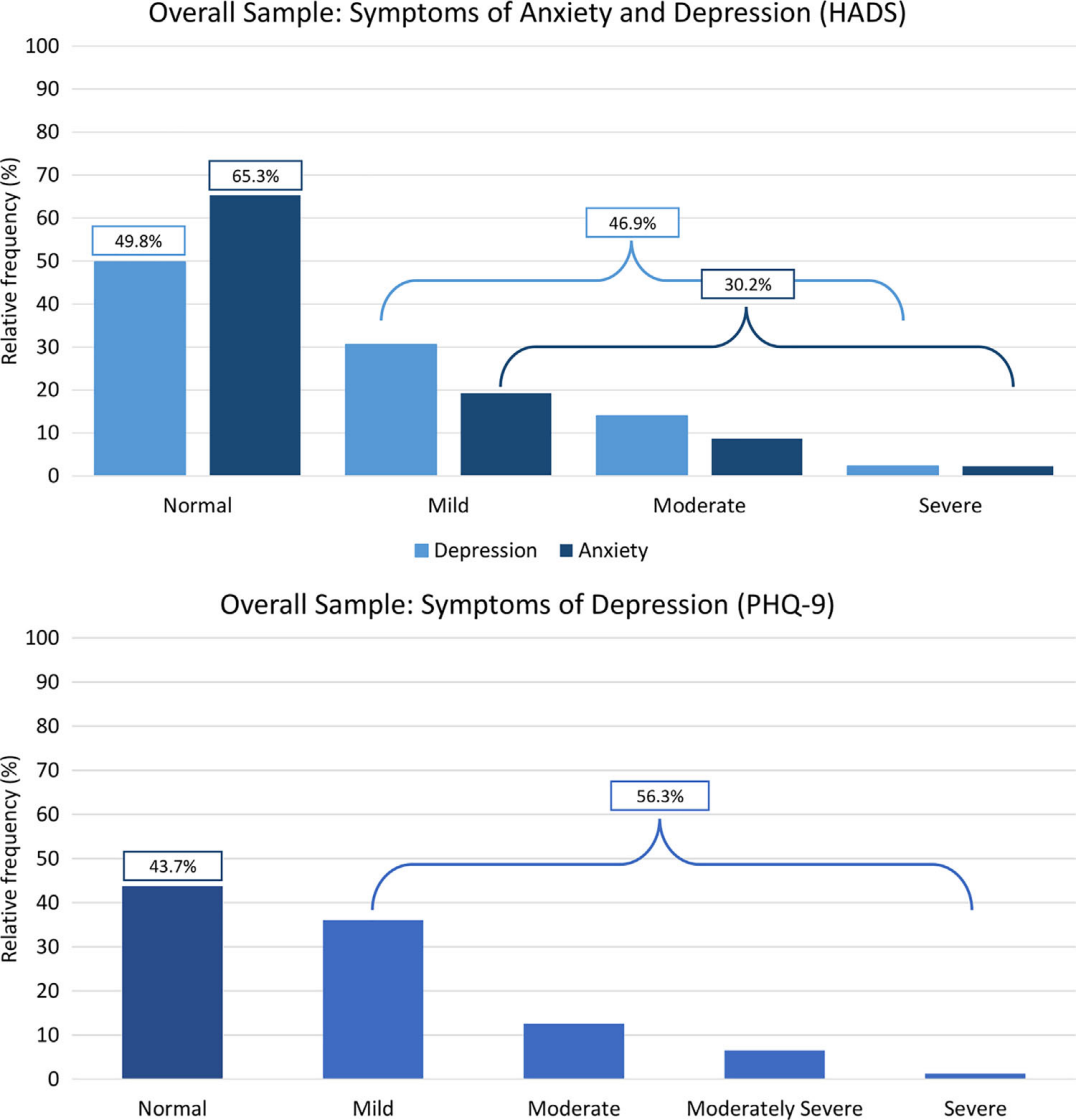
(**a**) HADS Score range: 0–7 = normal, 8–10 = mild, 11–14 = moderate, 15–21 = severe. *No group differences for heart‐ versus non‐heart‐related events for anxiety (*p* = 0.774) and depression (*p* = 0.565). (**b**) PHQ‐9 Score range: 0–4 = normal, 5–9 = mild, 10–14 = moderate, 15–19 = moderately severe, 20–27 = severe. *No group differences for heart‐ versus non‐heart‐related events for depression (*p* = 0.817).

Based on the HADS, for the overall sample symptoms of anxiety (30.2%) and depression (46.9%) (levels higher than 7) were reported (Figure 3a). Sub‐analyses based on mean (±SD) scores for those with versus without heart‐related events of PTSS revealed no significant group differences for anxiety (*p* = 0.774) and depression (*p* = 0.565). Depression symptoms assessed by the PHQ‐9 confirmed this observation (*p* = 0.817; Figure 3b).

#### 3.2.3 Health‐Related Quality of Life (HRQoL)

The KCCQ sum score revealed a mean (±SD) score of 63.2±17.7% for the overall sample. Sub‐analyses based on mean (±SD) scores for those with versus without heart‐related events of PTSS revealed no significant group differences for the KCCQ sum score (*p* = 0.226) (Table 3).

**TABLE 3:** HRQoL event‐type sub‐analysis.

Group	*Determinant*	*N*	Mean	SD	*T*	*df*	*p*‐value
Heart‐related event	KCCQ total score	57	62.61	18.96	−1.218	112	0.226
Non‐heart‐related event	KCCQ total score	57	66.75	17.28			

Abbreviations: df, degree of freedom; HRQoL, health‐related quality of life; KCCQ, Kansas city cardiomyopathy questionnaire; SD, standard deviation.

#### 3.2.4 Interrelationship Between Psychosocial Comorbid Conditions

The interrelation for the psychosocial comorbid conditions for the overall sample are outlined in Table 4. All psychosocial comorbid conditions showed statistically significant low to medium correlations.

**TABLE 4:** Interrelation matrix between psychosocial comorbid conditions.

	PTSS	HADS‐Anxiety	HADS‐Depression	PHQ‐9 Depression	KCCQ
PTSS	**1.000**				
HADS Anxiety	0.384^**^	**1.000**			
HADS Depression	0.430^**^	0.637^**^	**1.000**		
PHQ‐9 Depression	0.553^**^	0.669^**^	0.680^**^	**1.000**	
KCCQ	−0.385^**^	−0.548^**^	−0.621^**^	−0.694^**^	**1.000**

Abbreviations: HADS, hospital anxiety and depression scale; KCCQ, Kansas city cardiomyopathy questionnaire; PHQ‐9, patient health questionnaire 9‐item version; PTSS, posttraumatic stress symptoms (Breslau 7‐item scale)

** Pearson‐correlation significant at 0.01 level (2‐tailed).

### 3.3 Determinants of Psychosocial Comorbid Conditions

Mean comparisons by gender in those with non‐heart‐related events showed that women perceived significantly lower HRQoL (56.7% ± 18.4%) in comparison to men (68.9% ± 16.5%); mean difference −12.17 (95%CI: −23.89–0.45) (*T*(55) = −2.081; *p* = 0.042) (Table 5). Female gender was associated with an OR of 1.703 (95%CI: 1.217–2.382; *p* = 0.002) for HADS depression symptoms and for being on prescribed oral psychotropics (OR 6.623, 95%CI: 2.009–21.830; *p* = 0.002).

**TABLE 5:** HRQoL—gender and oral psychotropics sub‐analysis.

Group	Determinant	Sub‐group	*N*	Mean	SD	*T*	*df*	*p*‐value
**Gender**								
**Heart‐related event**	**KCCQ total score**	**Female**	6	63.68	17.60	0.145	55	0.885
		**Male**	51	62.49	19.28			
**Non‐heart‐related event**	**KCCQ total score**	**Female**	10	56.72	18.40	−2.081	55	**0.042**
		**Male**	47	68.89	16.46			
**Oral psychotropics**								
**Heart‐related event**	**KCCQ total score**	**yes**	4	56.40	1.45	−2.397	54	**0.020**
		**no**	53	63.08	19.59			
**Non‐heart‐related event**	**KCCQ total score**	**yes**	16	56.15	17.61	−3.108	55	**0.003**
		**no**	41	70.89	15.47			

HRQoL sub‐analysis for those taking prescribed oral psychotropics was significantly lower in comparison to the sub‐group not taking oral psychotropics (56.4% ± 1.4% vs. 63.1% ± 19.6%; *T*(55) = −2.397; *p* = 0.020) in patients who had experienced heart‐related events. Also, HRQoL sub‐analysis for subgroups on psychotropics (yes/ no) revealed significantly lower mean scores for those on psychotropics (56.1% ± 17.61% vs. 70.9% ± 19.6%; *T*(55) = −3.108; *p* = 0.003) for the non‐heart‐related event group (Table 5).

58.9% of the overall sample experienced an adverse event, defined as having experienced bleeding (10.9%), an infection of the driveline (31.3%) or a thromboembolic neurological event (7.9%) postimplantation. There were no significant differences identified between the groups with or without a reported heart‐related traumatic event (*p* = 0.850).

## 4 Discussion

Our study aimed to investigate the occurrence of PTSS in a large multicenter national trial of 393 patients on ongoing VAD support; the response rate for PTSS data was 67.4% (*n* = 265). The weighted mean prevalence for patients verbalizing any subjectively perceived a serious or traumatizing life event was 43.0% (95% CI 36.98%–49.22%). These events were categorized by content into events being non‐heart‐related (50%) versus those being heart‐related (50%). Heart surgery due to VAD implantation was reported most often as a “traumatizing life event”. Overall, 12.5% of the entire sample were on oral prescribed psychotropics, however this percentage was significantly higher in those with non‐heart‐related events in comparison to the sub‐sample with heart‐related events. Also, women were shown to have significantly higher prescription rates for psychotropics. Females reported significantly lower HRQoL in comparison to males for those with non‐heart‐related events.

In the non‐heart‐related event sub‐groups, our study revealed a significantly higher percentage of women being prescribed oral psychotropics and women reported on significantly lower HRQoL in comparison to men. This observation may lead to the clinically important conclusion that women should be given more psychosocial support while being on VAD support. A recently updated position statement recommends regular and continuous support of these patients by specially trained professionals [23]. However, no significant differences occurred for PTSS between genders, although estimates for lifetime PTSS in community samples [13] suggest that women face a higher likelihood of experiencing traumatizing events (10%–12%) in comparison to men (5%–6%) [13]. Although our sample was typical and representative for heart failure patients treated with VAD in concordance with the EUROMACS registry (2025) [24], it was not equally distributed with respect to gender, with women representing 12% of the overall sample. Based on the Breslau scale sum score, just one woman appeared to be symptomatic for PTSS. Traumatizing events reported in the open‐ended question format confirmed this finding. Overall, 16 women reported having experienced a traumatizing event, these included reports by experienced violence = 4; death/serious disease of a family member = 3; serious somatic disease [non‐heart, self] = 2; mental disease [self] = 1; heart surgery = 2; myocardial infarction = 1; defibrillation = 1; heart failure = 2; others = 1. However, this small sample size was not adequate for further statistical analysis. Analysis on the type of traumatizing event experienced (heart‐related/non‐heart‐related) revealed no statistically significant differences in regard to gender.

Symptomatic posttraumatic stress occurred in 3.7% of the overall sample assessed by the Breslau scale [18, 19], a standardized PRO measure. Given the fact that VAD patients were exposed to serious physical and psychosocial stressors, this proportion seems rather small. In comparison, a recently published estimate of PTSS of organ transplant recipients based on PRO assessments ranged between 0% and 46% [25]. The authors discussed that estimates depended on the assessment method and PRO instrument applied [25]. However, focusing in on heart transplant patients, PTSS symptoms were prevalent in 6%–14%, and in a US population, prevalence rates were as high as 3.5%, as reported by Davydow and associates [25]. Two recent studies [26, 27] assessed PTSS in VAD patients and reported estimates ranging between 23.5% and 27% [26, 27]. Both studies used a PRO approach; however comparability with our findings might be limited due to several PRO measures being utilized, samples being small and broad in the time post‐implant, respectively [26, 27].

Fifty‐seven patients (21.5%) of our sample were reported to carry memories of a subjectively perceived serious or traumatizing life event and verbalized a name for this event (e.g. death of a loved one or having had experienced or witnessed violence), which showed that these events were completely unrelated to their heart disease and subsequent VAD implantation. However, those precisely named events made it distinct to us as clinicians that patients carry their life experiences “in their backpacks” when entering our wards or outpatient clinics. Our study team was multiprofessional and included a trained psychologist or psychotherapist per participating site. During survey data analysis, participants with low psychosocial outcomes were reported to VAD‐Team psychologists on the study sites for further care. This was important, because although completely unrelated to the disease and its trajectory, those life experiences may hold potential to influence the patients’ outcome.

From a methodological standpoint, the capture mode for PTSS might have impacted, at least to some extent, the proportion of those who had experienced a traumatizing event and were identified as such in this sample. The German validated version of the 7‐item Breslau scale was applied. Patients were asked about (1) the occurrence of traumatic events, and (2) rating of perceived PTSS. According to Freedy and colleagues [13] this so‐called ‘non‐linkage’ strategy is thought to be more accurate, but tends to produce somewhat higher PTSS rates. Diagnostic efficiency was adequate (AUC >0.80) for the Breslau scale in a larger community drawn sample [13]. However, diagnostic efficiency studies testing psychometric properties of existing PTSS screening measures available for general populations need to be verified to be reliable, valid, and sensitive for patients while on VAD support. The fact that a very low negative correlation between the PTSS sum score and the KCCQ sum score was identified can be interpreted cautiously and conservatively that divergent construct validity can be accepted. Correlations between other psychosocial conditions were in line with clinical expectations.

Anxiety and depression were prevalent in our sample; however, they were unrelated to heart‐related events of PTSS. Likewise, Weerahandi and associates [28] assessed the relationship between psychosocial symptoms, namely anxiety, depression, and posttraumatic stress and VAD implantation in a prospective multicenter cohort study over a 48‐week period following VAD implant. Their findings suggest that VAD implant did not induce depression, anxiety or PTSS, but rather VAD implantation may have had a positive impact on depression, anxiety, and PTSS by providing hope for an at least temporarily reversed illness trajectory. Our findings were cross‐sectional and thus single point in time. However, since PTSS screening using the 7‐item Breslau scale was as low as 3.7%, we might have seen this reversed effect as a result of the VAD implantation as well. Future prospective, longitudinal studies that can systematically capture the timing of cardiac interventions, gender‐specific trauma exposure and transplantation candidacy are needed to confirm this effect.

## 5 Strengths and Limitations

This study was a large, national, multicenter study. Assessments for psychosocial comorbidities were based on standardized validated PRO measures. First, these measures were generic by nature and may have missed VAD‐specific symptomatology and thus, may have underestimated the outcome measures under investigation to some extent. Additionally, the open‐ended question lacked a temporal context for reported events. Second, data collection utilized the paper‐pencil method, and was performed by trained staff at all four study centers simultaneously. Staff were trained to administer the instruments at a workshop, which aimed to standardize the data collection procedures. In addition, regular virtual meetings were scheduled during the entire data collection process to allow for exchange of experiences and discussion of any questions or concerns, which may have occurred at a specific center. Social desirability cannot be completely ignored, although no center‐related biases were identified. Third, the data collection method may have resulted in a moderate response rate of 67.4%. Patients on VAD support are aware of the fact that psychosocial comorbidities may delay or even exclude them from being placed on the waitlist for heart transplant or device exchange, thus they may have withheld their condition related to having experienced traumatizing events and their effects on their psychosocial well‐being. Fourth, being typical and representative for heart failure patients, our sample was not equally distributed with respect to gender. Women comprised 12% of the overall sample. Fifth, this being a cross‐sectional study, we cannot determine whether the traumatizing event might have been a trigger for the development and/ or progression of the end‐stage heart disease or whether the heart disease itself, including its implicit “life‐threat” and the invasive diagnostic and therapeutic procedure (*s*) might have served as a trigger for trauma and PTSS symptoms. Sixth, it can be concluded, albeit with caution, that patients with previous heart procedures might eventually confound the interpretation of the development of PTSS symptoms after VAD.

## 6 Conclusion

To the best of our knowledge, this is one of the first studies systematically assessing PTSS in a large multicenter national trial of 265 patients on ongoing VAD support. A relevant percentage of 43% of patients reported to have experienced subjectively perceived serious or traumatizing life events. Most importantly, heart surgery due to VAD implantation was reported most often as a “traumatizing life event”, although heart‐related events did not result in higher psychosocial comorbidities nor in higher symptoms of PTSS, leading to the careful conclusion that VAD implantation is a life‐saving procedure with no increased risk for causing trauma. Nevertheless, a significant proportion of patients did bring experiences of lifetime trauma events “in their backpacks” and may warrant further screening. Women were more likely to be on oral prescribed psychotropics and reported significantly lower HRQoL in comparison to men for those with non‐heart‐related events. Thus, to point to the introductory question: posttraumatic stress in patients on VAD support is a relevant syndrome that warrants attention from clinicians and researchers. Psychosocial outcome requires routine clinical screening and interventions in symptomatic patients following VAD implantation.

## Author Contributions

Christiane Kugler served as grant holder and coordinator for this study, she contributed to the study design, coordination of data acquisition, analysis and interpretation, and manuscript writing. Sadhbh Byrne contributed to the data analysis and manuscript preparation. Hannah Spielmann contributed to the study design, data acquisition and manuscript preparation. Fabian Richter contributed to data acquisition, and manuscript preparation. Sandra Semmig‐Könze contributed to site coordination, data acquisition, and manuscript preparation. Christine Spitz‐Koeberich contributed to site coordination, data acquisition, and manuscript preparation. Wolfgang Albert contributed to site coordination, data acquisition, and manuscript preparation. Katharina Tigges‐Limmer contributed to grant application, study design, interpretation of the results, and manuscript preparation.

## Conflicts of Interest

The authors declare no conflicts of interest.

## Data Availability

The data that support the findings of this study are available on request from the corresponding author. The data are not publicly available due to privacy or ethical restrictions.
